# Supplementary material for: The effect of intrapartum prolonged oxygen exposure on fetal metabolic status: secondary analysis from a randomized controlled trial
Source: Front Endocrinol (Lausanne). 2023 Jun 27;14:1204956. doi: 10.3389/fendo.2023.1204956 (PMC10335765; doi:10.3389/fendo.2023.1204956)
Supplement: Supplementary file 3 [file DataSheet_3.zip › Result-X101SC21092977-Z01-J001-B1-42/Met_results_dir.html]

Directory Tree


# Directory Tree

../results
  
 ├── 1.MetQuant-QC:代谢物质控结果目录
  
 ├── 2.MetAnnotation:代谢物注释结果目录
  
 │ ├── KEGG
  
 │ ├── HMDB
  
 │ ├── Lipidmaps
  
 │
  
 ├── 3.MetDiffScreening:代谢物定量结果目录
  
 │ ├── O.vs.A
  
 │
  
 ├── 4.MetDiffAnalysis:差异代谢物分析结果目录
  
 │ ├── O.vs.A
  
 │
  
 ├── 5.MetKeggEnrichment:差异代谢物Kegg富集分析结果目录
  
 │ ├── O.vs.A

---

tree v1.5.3 © 1996 - 2009 by Steve Baker and Thomas Moore   
HTML output hacked and copyleft © 1998 by Francesc Rocher   
Charsets / OS/2 support © 2001 by Kyosuke Tokoro
